# A novel approach for repair of right sided congenital diaphragmatic hernia and hepatopulmonary fusion

**DOI:** 10.1093/jscr/rjae566

**Published:** 2024-09-05

**Authors:** Caitlin J Cain-Trivette, Newell Bryce Robinson, Angela Kadenhe-Chiweshe, Shaun Steigman, Nitsana A Spigland

**Affiliations:** Department of Surgery, The New York Hospital-Cornell Medical Center, 525 E 68th Street New York, NY 10065, United States; Department of Surgery, The New York Hospital-Cornell Medical Center, 525 E 68th Street New York, NY 10065, United States; Department of Pediatric Surgery, The New York Hospital-Cornell Medical Center, 525 E 68th Street New York, NY 10065, United States; Department of Pediatric Surgery, Good Samaritan Hospital Medical Center, 1000 Montauk Hwy, West Islip, NY 11795, United States; Department of Pediatric Surgery, The New York Hospital-Cornell Medical Center, 525 E 68th Street New York, NY 10065, United States

**Keywords:** hepatopulmonary fusion, congenital diaphragmatic hernia, case report

## Abstract

Right sided congenital diaphragmatic hernia (CDH) associated with hepatopulmonary fusion (HPF) is a rare congenital anomaly in which the herniated liver is fused with lung parenchyma. We discuss the case of an infant with right-sided CDH and HPF found on index operation for repair of right-sided CDH. Due to the high incidence of vascular anomalies associated with HPF the decision was made to close the patient and get further imaging to characterize the HPF before returning to the operating room for definitive repair. We describe a novel and successful operative approach where the liver was left entirely fused to the lung and the liver was partially brought down from the chest and was plicated to the diaphragm form a seal between the parietal and pleural cavities to repair the CDH.

## Introduction

Congenital diaphragmatic hernia (CDH) occurs in one in 2500–3500 live births, when the diaphragm fails to close during fetal development. It is often detected with prenatal screening and typically affects the left side of the diaphragm, but in ~17% of cases, it can occur on the right side [1]. Hepatopulmonary fusion (HPF) occurs in a subset of patients with right sided CDH. In HPF the fusion of the liver and lung tissue results in abnormal patterns of blood flow and gas exchange, which in addition to pulmonary hypoplasia, is associated with pulmonary hypertension leading to poor outcomes with ~50% survival rate [2]. We describe the clinical presentation, imaging findings, and recommended management of this condition. We highlight the importance of recognizing HPF and its associated congenital anomalies and strategies for operative planning.

## Case report

The patient is a female infant born at 38 weeks and 5 days gestation with a birthweight of 2980 grams and Apgar scores of five and eight at 1 and 5 minutes, respectively. Prenatal testing was normal except for a low first trimester PAPP-A test. A detailed sonogram at 20 weeks was normal and a follow up at 37 weeks showed a borderline dilated right atrium and trace tricuspid regurgitation. There were no signs of polyhydramnios throughout the pregnancy.

**Figure 1:**
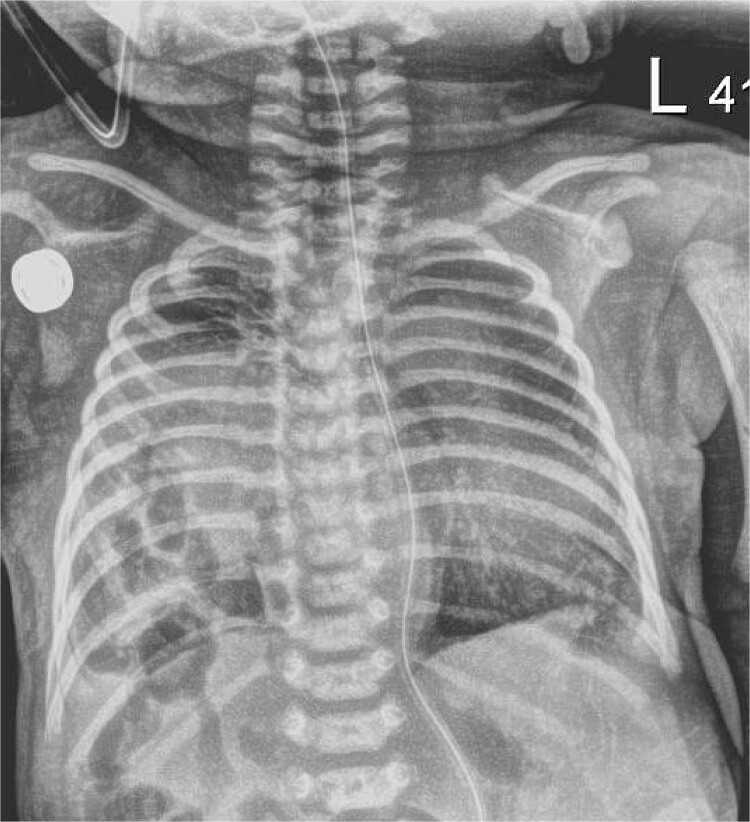
Chest X-ray, hour of life 1: there is a lucency in the right chest from the congenital diaphragmatic hernia as well as loops of bowel. There is no evidence of mediastinal shift and the superior aspect of the lung appears to aerate.

The infant presented with respiratory distress within one hour of life and underwent intubation. A chest X-ray showed a right sided CDH with bowel in the right chest and no evidence of mediastinal shift (Fig. 1). An echocardiogram showed mild dilation of the right atrium and right ventricle with moderately increased pulmonary artery pressures with a large PDA and PFO without flow reversal or left to right shunting. The initial echo showed three pulmonary veins with two left sided veins draining into the left atrium and a right upper pulmonary vein draining into the left atrium.

The patient was taken to the operating room on DOL3 for definitive repair via right subcostal laparotomy. The bowel was reduced into the abdomen and the patient was noted to have a HPF with a portion of the right lobe of the liver in the right chest which could not be reduced. The procedure was aborted so additional imaging could be obtained to further delineate the anatomy with planned re-exploration.

**Figure 2:**
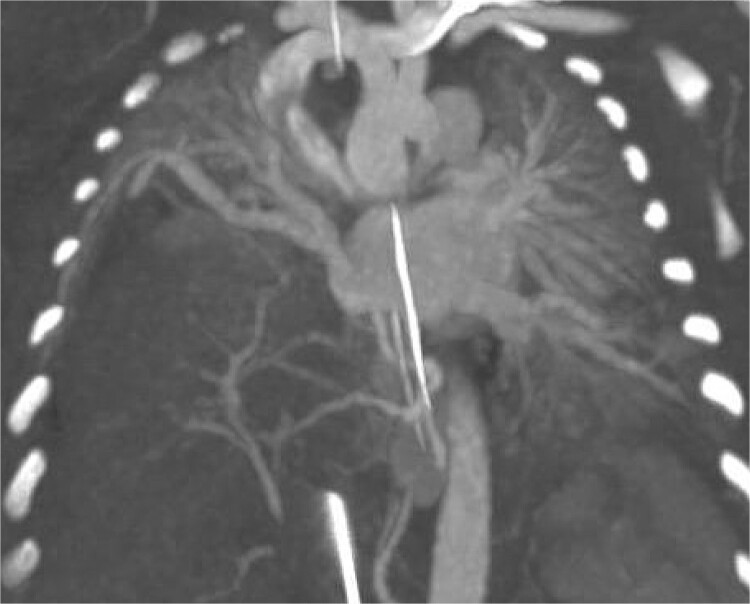
CT arteriography: coronal slice demonstrating anatomy of pulmonary veins with two left sided pulmonary veins draining into the left atrium and the single right sided upper pulmonary vein also draining into the left atrium.

Postoperatively the patient developed worsening pulmonary hypertension and was started on milrinone. Subsequent imaging included an MRI which demonstrated a right sided CDH and a portion of the right hepatic lobe in the chest. Because of difficulty differentiating the vasculature on MRI, a CTA was performed confirming the presence of the HPF with a right sided intra-lobar sequestration and a single right pulmonary vein (Fig. 2).

**Figure 3:**
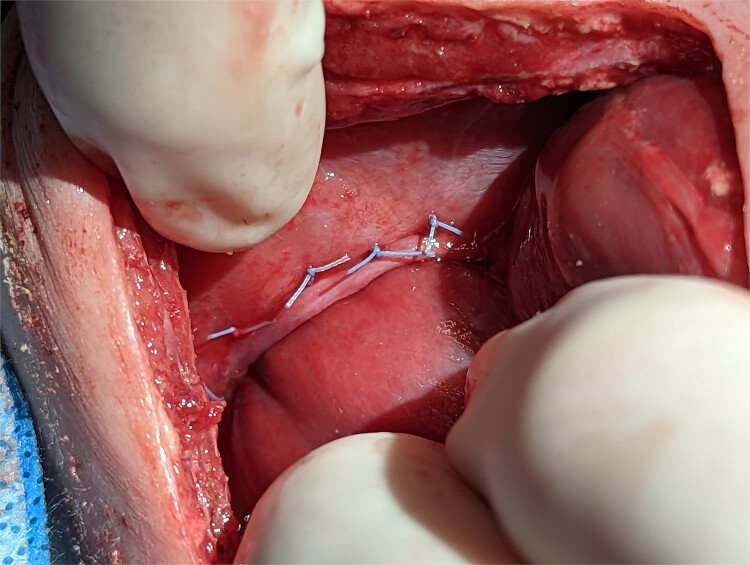
Intraoperative technique: the liver is plicated to the diaphragm with interrupted horizontal mattress sutures creating a seal between the thoracic and intra-abdominal cavities.

On post operative day 11 the patient was taken back to the operating room for definitive repair. The right main pulmonary vein coursed immediately adjacent and superior to the HPF. The operative plan was to forgo separation of the HPF and close the diaphragmatic defect around the liver, leaving the HPF in situ. The diaphragm was sewn to the liver with a series of horizontal mattress sutures which provided an effective seal between the peritoneal and pleural cavities (Fig. 3).

The patient’s postoperative course was complicated by ongoing pulmonary hypertension. On DOL31 the patient was extubated. The patient was subsequently discharged from the hospital on DOL138.

## Discussion

HPF remains an uncommon congenital anomaly that is clinically challenging to manage. A study of 37 patients from 2019 which looked at all cases of HPF from 1977 onward showed multiple strategies for operative management of HPF including: complete, partial, or no separation of liver and lung, partial hepatic or pulmonic resection, and partial or complete diaphragm repair with or without patch. What stood out from this study is that most children who died did so in the immediate postoperative period (within 4 days) suggesting there was a surgical complication which led to their demise and that there are operative pitfalls to avoid [2].

In patients with HPF and a right sided CDH, the incidence of aberrant vascular anatomy is up to 83%; this can make separation difficult, morbid, and fatal [2]. The optimal operative approach requires pre-operative consideration of the patient’s vascular anatomy which can be delineated with pre-operative imaging such as CT-angiography. Given the potential for intra-operative vascular catastrophe, surgeons should not attempt complete separation of a HPF. There have been good outcomes with partial lateral separation leaving the medial fusion intact with a diaphragmatic patch or primary repair, or as in with our case no attempted separation with plication of the liver capsule to the diaphragm.

The operative approach our team chose leads to the fundamental question, what is the objective of CDH repair. First, it is to prevent bowel herniation, which in our case was accomplished by the liver plugging the defect; however, it is unclear how much this aided diaphragmatic movement and pulmonary function. Surgeons are willing to sacrifice diaphragm movement in cases of simple CDH when a patch repair is necessary, and in terms of aiding pulmonary function, the true benefit of CDH repair is not having bowel in the pleural cavity competing for space — particularly as the number and size of alveoli increase until the lung fully matures in young adulthood [3]^.^

It seems unlikely that completing a true diaphragm repair makes a significant difference in patient outcomes weighed with the consequences of lung/liver separation in cases of HPF. This is especially true as a patient’s underlying pulmonary hypoplasia and their degree of concomitant hypoplasia of the pulmonary vasculature with associated pulmonary hypertension and right ventricular dysfunction is mutually exclusive from the issue of HPF, and in CDH the degree of pulmonary hypertension is an independent predictor of mortality [4]^.^ In the safest operative approach, the surgeon should not fixate on complete separation of the fusion, rather they should focus on safely reducing bowel contents into the abdomen and repairing or plugging the diaphragmatic defect while being mindful of aberrant vascular anatomy.
